# Circulating histone H3 levels in septic patients are associated with coagulopathy, multiple organ failure, and death: a single-center observational study

**DOI:** 10.1186/s12959-018-0190-4

**Published:** 2019-01-14

**Authors:** Yayoi Yokoyama, Takashi Ito, Tomotsugu Yasuda, Hiroaki Furubeppu, Chinatsu Kamikokuryo, Shingo Yamada, Ikuro Maruyama, Yasuyuki Kakihana

**Affiliations:** 1 Department of Emergency and Intensive Care Medicine, Kagoshima University Graduate School of Medical and Dental Sciences, 8-35-1 Sakuragaoka, Kagoshima, 890-8544 Japan; 2 Department of Systems Biology in Thromboregulation, Kagoshima University Graduate School of Medical and Dental Sciences, Kagoshima, Japan; 3 R&D Center, Shino-Test Corporation, Sagamihara, Japan

**Keywords:** Histone, Sepsis, Coagulopathy, Organ failure

## Abstract

**Background:**

Nuclear histone proteins are released into the extracellular space, and act as major mediators of coagulopathy and remote organ failure in septic animals. However, the circulating histone levels in septic patients have not been precisely quantified.

**Methods:**

Using a novel enzyme-linked immunosorbent assay for histone H3 detection, we measured the serum histone H3 levels in 85 patients admitted to the intensive care unit because of infectious diseases. We then evaluated the associations of circulating histone H3 levels with organ failure, coagulopathy, and mortality.

**Results:**

Circulating histone H3 levels were significantly higher in patients with coagulopathy, and were positively correlated with numbers of organ failures. Circulating histone H3 levels were also associated with fatal outcome. Receiver-operating characteristic analyses revealed that the predictive performance of circulating histone H3 levels for mortality was higher than that of conventional inflammatory markers, including white blood cell count, C-reactive protein, and cell-free DNA.

**Conclusions:**

Circulating histone H3 levels are associated with coagulopathy, multiple organ failure, and death in patients requiring intensive care because of infectious diseases.

## Background

Sepsis is defined as life-threatening organ dysfunction caused by a dysregulated host response to infection [1]. Although the dysregulated host response has not been clarified in detail, an overwhelming inflammatory response is considered to be involved. Nevertheless, anti-inflammatory agents, such as tumor necrosis factor antagonists and interleukin-1 receptor antagonists, did not improve the survival of patients with sepsis [2].

Recent studies have suggested that extracellular histones are potential mediators of death in sepsis [3, 4]. Histones, the most abundant proteins in the nucleus, comprise linker histone H1 and core histones H2A, H2B, H3, and H4. In animal models of sepsis, histones are released into the extracellular space [3, 5] where they induce platelet aggregation [6, 7], endothelial damage [3, 8], and cardiac injury [5, 9]. These findings have led to increased demand for convenient and reproducible methods to measure circulating histone levels. However, there are no reliable methods for quantitative analysis of circulating histone levels other than semiquantitative western blot analysis [3, 5, 10]. In the present study, we quantified serum histone H3 levels using a newly developed enzyme-linked immunosorbent assay (ELISA) and found that circulating histone H3 levels are associated with coagulopathy, multiple organ failure, and death in septic patients.

## Materials and methods

### Patients

This was a single-center observational study. Between July 2015 and June 2016, 85 patients with suspected infections were admitted to the intensive care unit (ICU) at Kagoshima University. Written informed consent was obtained from all patients for using residual blood samples of routine blood tests for research. Residual serum samples from the patients were anonymized and used for measurements of histone H3 and cell-free DNA. Clinical data, including Systemic Inflammatory Response Syndrome (SIRS) score, Acute Physiology and Chronic Health Evaluation II (APACHE II) score without the Glasgow Coma Scale component, and Sequential Organ Failure Assessment (SOFA) score without the Glasgow Coma Scale component, were also anonymized and analyzed. Organ failure was defined as a score of ≥3 for each component of the SOFA score [11]. Shock was defined as a score of ≥2 for the cardiovascular component of the SOFA score combined with a lactate level of > 2 mmol/L. Diagnosis of disseminated intravascular coagulation (DIC) was made according to the criteria established by the Japanese Association for Acute Medicine [12]. The study was compliant with the Declaration of Helsinki and approved by the Ethics Committee of Kagoshima University.

### Measurement of histone H3 levels

Serum histone H3 levels were measured as recently described [9]. Briefly, polystyrene microtiter plates (Nunc, Roskilde, Denmark) were coated with 100 μL of 1 mg/L anti-histone H3 antibody (Shino-Test Corporation, Sagamihara, Japan). After washing and blocking, the plates were incubated with 100 μL of diluted calibrator and serum samples for 24 h at room temperature. After washing, the plates were incubated with 100 μL of anti-histone H3 peroxidase-conjugated antibody (Shino-Test Corporation) for 2 h at room temperature. The plates were washed again, and incubated with the chromogenic substrate 3,3′,5,5′-tetra-methylbenzidine (Dojindo Laboratories, Kumamoto, Japan). The reaction was terminated with 0.35 mol/L Na_2_SO_4_, and the absorbance at 450 nm was measured with a microplate reader (Model 680; Bio-Rad, Hercules, CA). A standard curve was obtained with purified calf thymus histone H3 (Roche, Stockholm, Sweden). The lower detection limit of the ELISA was 2 ng/mL, and linearity was observed in the range up to 250 ng/mL. The ELISA specifically detected histone H3, and did not react with other histone family proteins, including histone H2A, H2B and H4 [9].

### Measurement of serum cell-free DNA levels

Cell-free DNA levels in serum samples were quantified as described [13]. Briefly, diluted serum samples (1:10 dilution in PBS) were mixed with SytoxGreen (2 μM; Molecular Probes, Eugene, OR). Fluorescence was recorded by an Infinite M200 fluorometer (Tecan, Männedorf, Switzerland) with 485 nm excitation and 538 nm emission. Autofluorescence was determined in samples without SytoxGreen and defined as background fluorescence. DNA concentrations were calculated based on a standard curve of known DNA concentrations.

### Measurement of blood cell counts and C-reactive protein levels

Complete blood counts were performed using an automated counting device (XE-5000; Sysmex Corporation, Kobe, Japan). Serum C-reactive protein (CRP) levels were examined with a BioMajesty JCA-BM6070 (Jeol Ltd., Tokyo, Japan).

### Statistical analysis

Circulating histone H3 levels are shown as median (lower quartile–upper quartile) or box plot with lower extreme, lower quartile, median, upper quartile, and upper extreme values, as well as outliers. Circulating histone H3 levels in patients with multiple organ failure were compared with those in patients without organ failure by the Shirley–Williams test. Circulating histone H3 levels, cell-free DNA levels, CRP levels, and white blood cell counts in 28-day survivors were compared with those in non-survivors by the Mann–Whitney *U* test. Receiver-operating characteristics (ROC) curve analysis with area under the curve (AUC) calculation was used to quantify the predictive performance of each parameter for 28-day mortality. A *p*-value of less than 0.05 was considered statistically significant.

## Results

### Baseline characteristics of the patients

The baseline characteristics of the 85 patients are shown in Table 1. All 85 patients had a SOFA score of ≥2, and 10 patients (15.7%) were SIRS-negative. Forty patients (47.1%) were associated with shock and 12 patients (14.1%) had died by day 28.

**Table 1:** Basic characteristics of the study patients

	All	Survivors (*n* = 73)	Non-survivors (*n* = 12)	*p*
Age	71 (24–92)	71 (*n* = 73)	72.5 (*n* = 12)	0.64
APACHE II	19 (7–33)	18.5 (*n* = 72)	22 (*n* = 12)	0.09
SOFA score	8 (3–17)	7 (*n* = 68)	11 (*n* = 11)	< 0.01
SIRS score	3 (0–4)	3 (*n* = 72)	3 (*n* = 12)	0.72
DIC score	4 (1–8)	4 (*n* = 65)	6 (n = 12)	0.06
Shock (%)	47.1	42.5	75	0.06

*APACHE II* Acute Physiology and Chronic Health Evaluation II without the Glasgow Coma Scale component, *SOFA* Sequential Organ Failure Assessment without the Glasgow Coma Scale component, *SIRS* Systemic Inflammatory Response Syndrome, *DIC* Disseminated Intravascular Coagulation;

Data for all subjects are shown as median (minimum–maximum), and data for survivors and non-survivors are shown as median (number of patients analyzed)

Differences between survivors and non-survivors were analyzed by the Mann–Whitney *U* test, except for shock (%), which was analyzed by the chi-square test

### Circulating histone H3 levels are associated with multiple organ failure

**Fig. 1:**
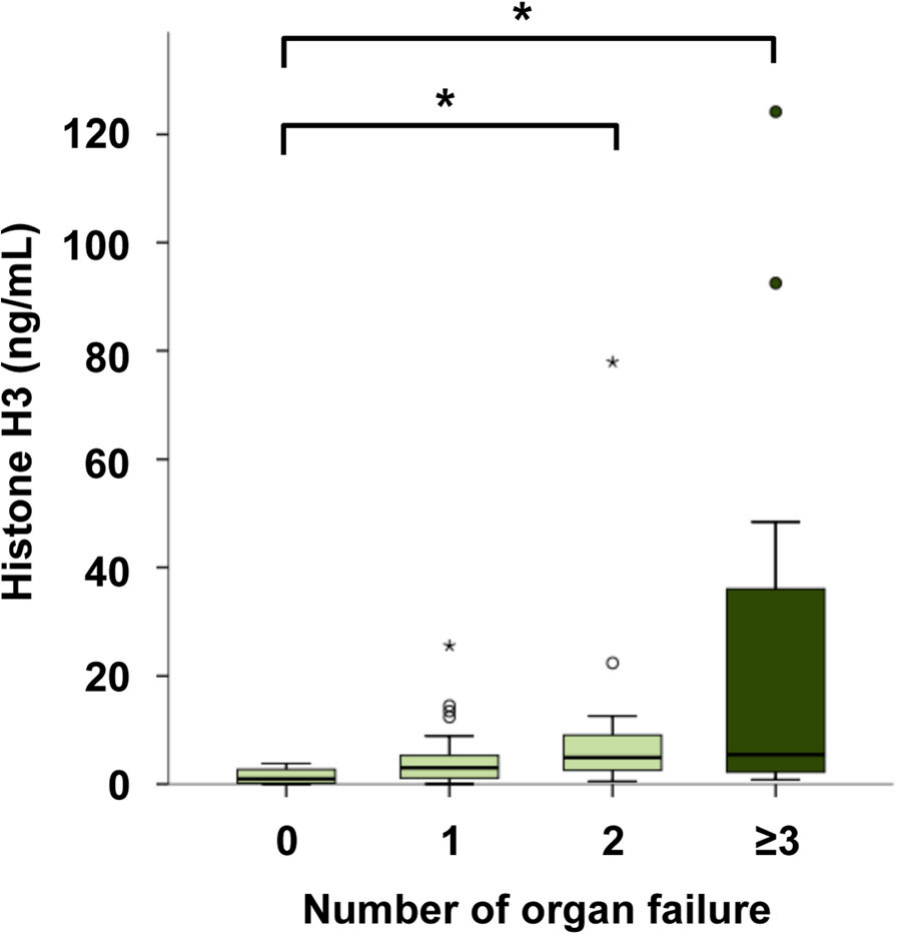
Circulating histone H3 levels are associated with multiple organ failure. Serum histone H3 levels within 24 h of ICU admission (day 1) were analyzed in septic patients. The septic patients were divided into four groups: those without organ failure (*n* = 5), those with one organ failure (*n* = 38), those with two organ failures (*n* = 27), and those with three or more organ failures (*n* = 14 for three organ failures; *n* = 1 for four organ failures). Data for four patients were missing because serum samples were not obtained within 24 h of ICU admission. Differences between serum histone H3 levels in patients with and without organ failure were analyzed by the Shirley–Williams test. *p <  0.05 versus patients without organ failure

Using a newly developed ELISA for histone H3 detection, we measured the circulating histone H3 levels in the 85 patients with infectious diseases. Serum histone H3 levels in patients without organ failure were 1.0 (0.2–2.7) ng/mL, while those in patients with one, two, and three or more organ failures were 3.0 (1.1–5.3), 4.9 (2.5–9.2), and 5.5 (2.2–36.1) ng/mL, respectively (Fig. 1). Serum histone H3 levels in patients with multiple organ failure were significantly higher than those in patients without organ failure (*p* <  0.05).

### Circulating histone H3 levels are associated with coagulation failure

**Fig. 2:**
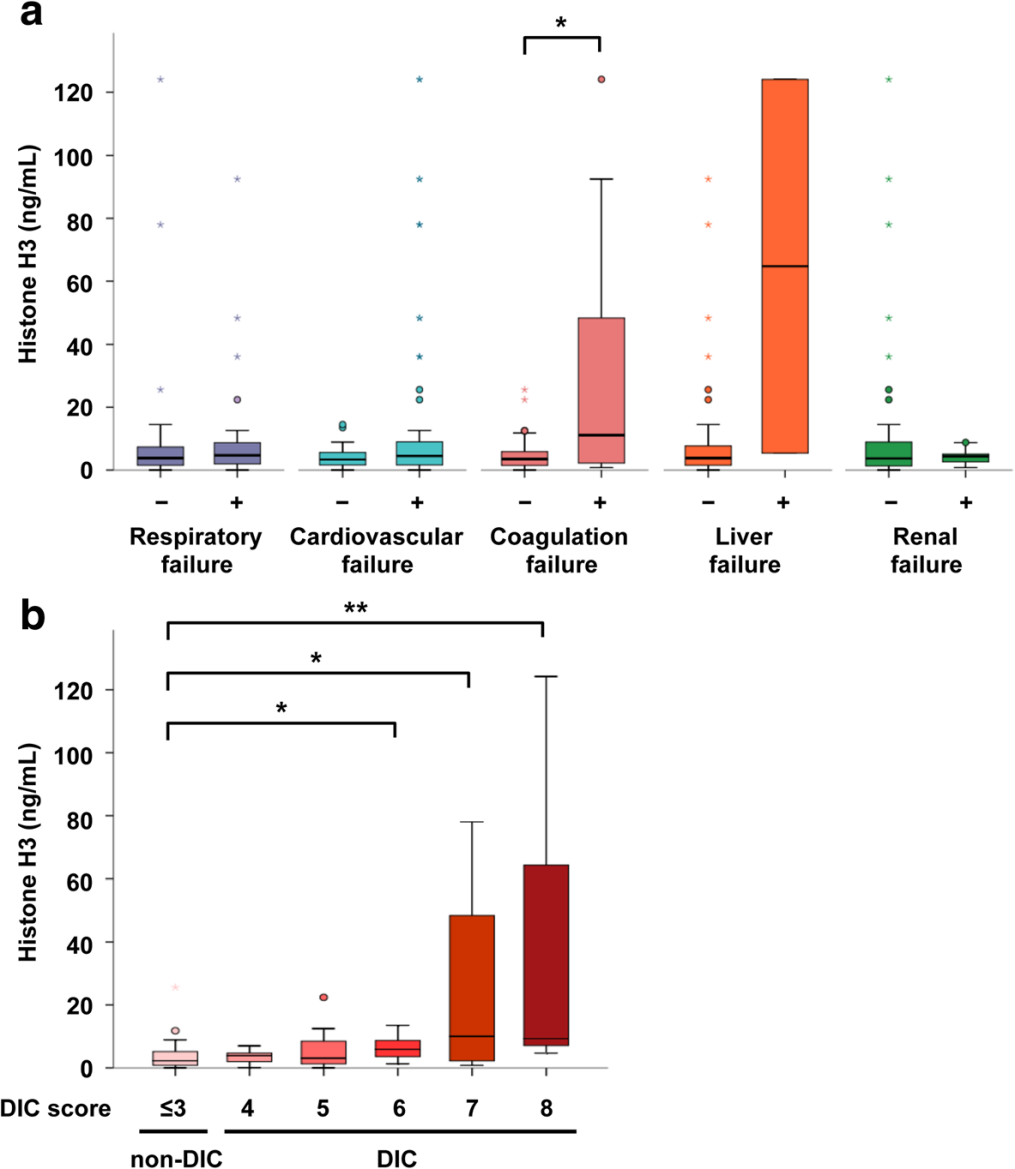
Circulating histone H3 levels are associated with coagulation failure. **a** Serum histone H3 levels within 24 h of ICU admission (day 1) were analyzed in septic patients with and without respiratory failure (*n* = 47 and *n* = 31, respectively), cardiovascular failure (*n* = 51 and *n* = 30, respectively), coagulation failure (*n* = 14 and *n* = 67, respectively), liver failure (*n* = 2 and *n* = 79, respectively), and renal failure (*n* = 19 and *n* = 62, respectively). Data for four patients were missing because serum samples were not obtained within 24 h of ICU admission. Differences between serum histone H3 levels in patients with and without each organ failure were analyzed by the Mann–Whitney *U* test. *p < 0.05 versus patients without organ failure. **b** Serum histone H3 levels within 24 h of ICU admission (day 1) were analyzed in septic patients. The septic patients were divided into six groups: those without DIC (*n* = 32), those with DIC score of 4 (*n* = 9), DIC score of 5 (*n* = 11), DIC score of 6 (*n* = 9), DIC score of 7 (*n* = 6), and DIC score of 8 (*n* = 7). Data for 11 patients were missing because serum samples were not obtained within 24 h of ICU admission or DIC scores were not available. Differences of serum histone H3 levels between patients with and without DIC were analyzed by the Shirley–Williams test. **p < 0.01 and *p < 0.05 versus patients without DIC

We evaluated the serum histone H3 levels in patients with each organ failure in the SOFA score, including respiratory, cardiovascular, coagulation, liver, and renal failures (Fig. 2a). Serum histone H3 levels in patients with coagulation failure (11.1 [2.2–48.4] ng/mL) were significantly higher than those in patients without coagulation failure (3.5 [1.5–5.9] ng/mL). Serum histone H3 levels did not differ significantly between patients with (4.5 [1.6–9.2] ng/mL) and without (3.4 [1.6–5.6] ng/mL) cardiovascular failure. However, extremely high histone H3 levels, including those exceeding 20 ng/mL, were associated with cardiovascular failure. It remained unclear whether serum histone H3 levels in patients with liver failure were higher than those in patients without liver failure because only two patients had liver failure on day 1. Serum histone H3 levels did not differ significantly between patients with and without respiratory failure or renal failure. These results indicate that circulating histone H3 levels are associated with organ failure, especially coagulation failure.

In the assessment of SOFA, coagulation failure is defined as the platelet count of less than 50,000/μL [11]. Since thrombocytopenia represents only a part of coagulation failure, we then assessed the status of DIC. Among 77 patients whose DIC scores on Day 1 were available, 43 patients were defined as a state of DIC. Serum histone H3 levels in patients with DIC (4.8 [2.2–9.9] ng/mL) were significantly higher than those in patients without DIC (2.3 [0.9–5.2] ng/mL). In particular, patients with a DIC score of ≥6 showed significantly high serum histone H3 levels (Fig. 2b). These findings indicate that circulating histone H3 levels are associated with a state of DIC.

### Circulating histone H3 levels are associated with fatal outcome

**Fig. 3:**
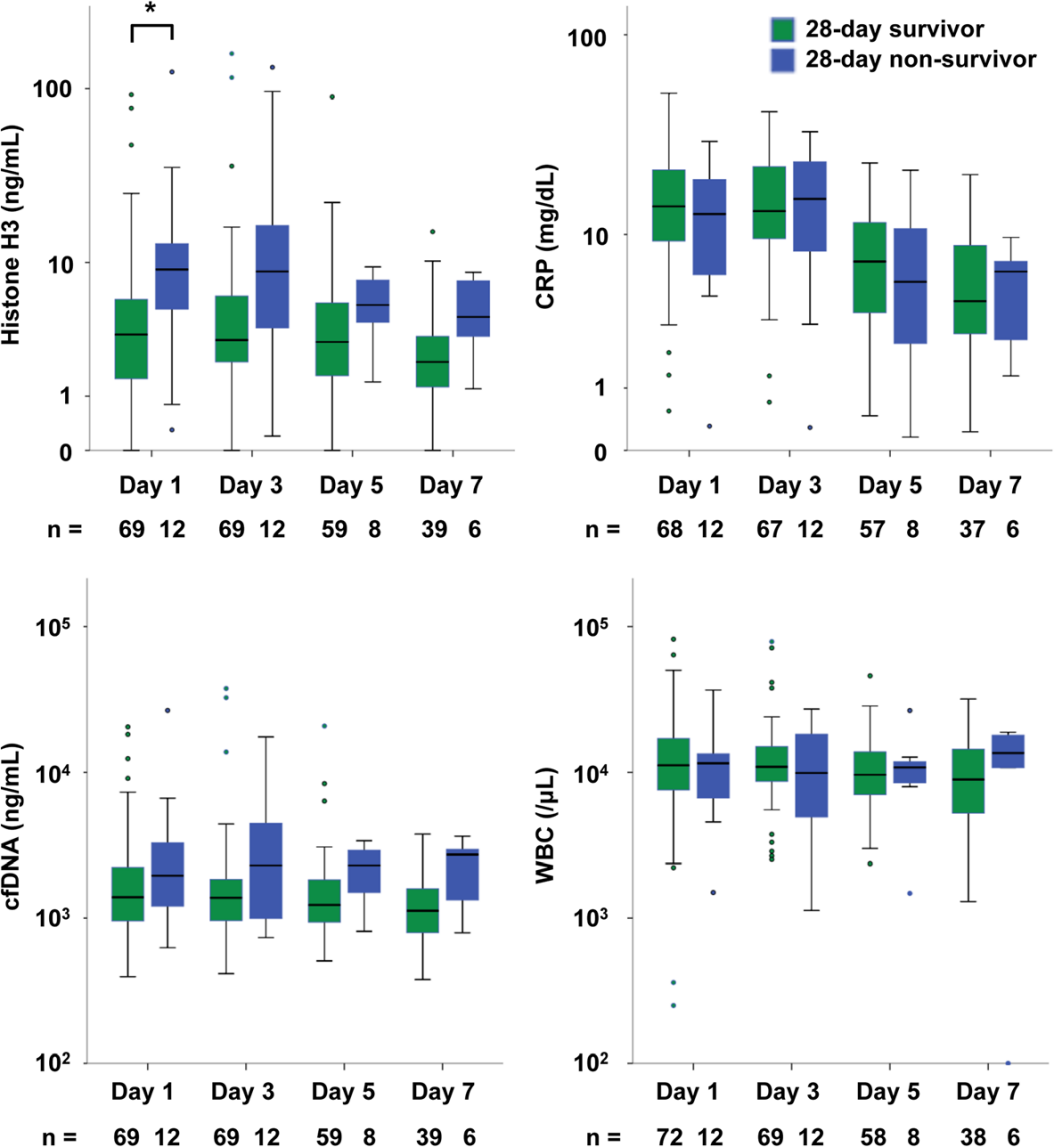
Circulating histone H3 levels are associated with fatal outcome. Serum histone H3 levels, cell-free DNA (cfDNA) levels, CRP levels, and white blood cell (WBC) counts on days 1, 3, 5, and 7 were analyzed. Patients who left the ICU were not followed up even if they survived. Differences between 28-day survivors and non-survivors were analyzed by the Mann–Whitney *U* test. *p < 0.05

**Fig. 4:**
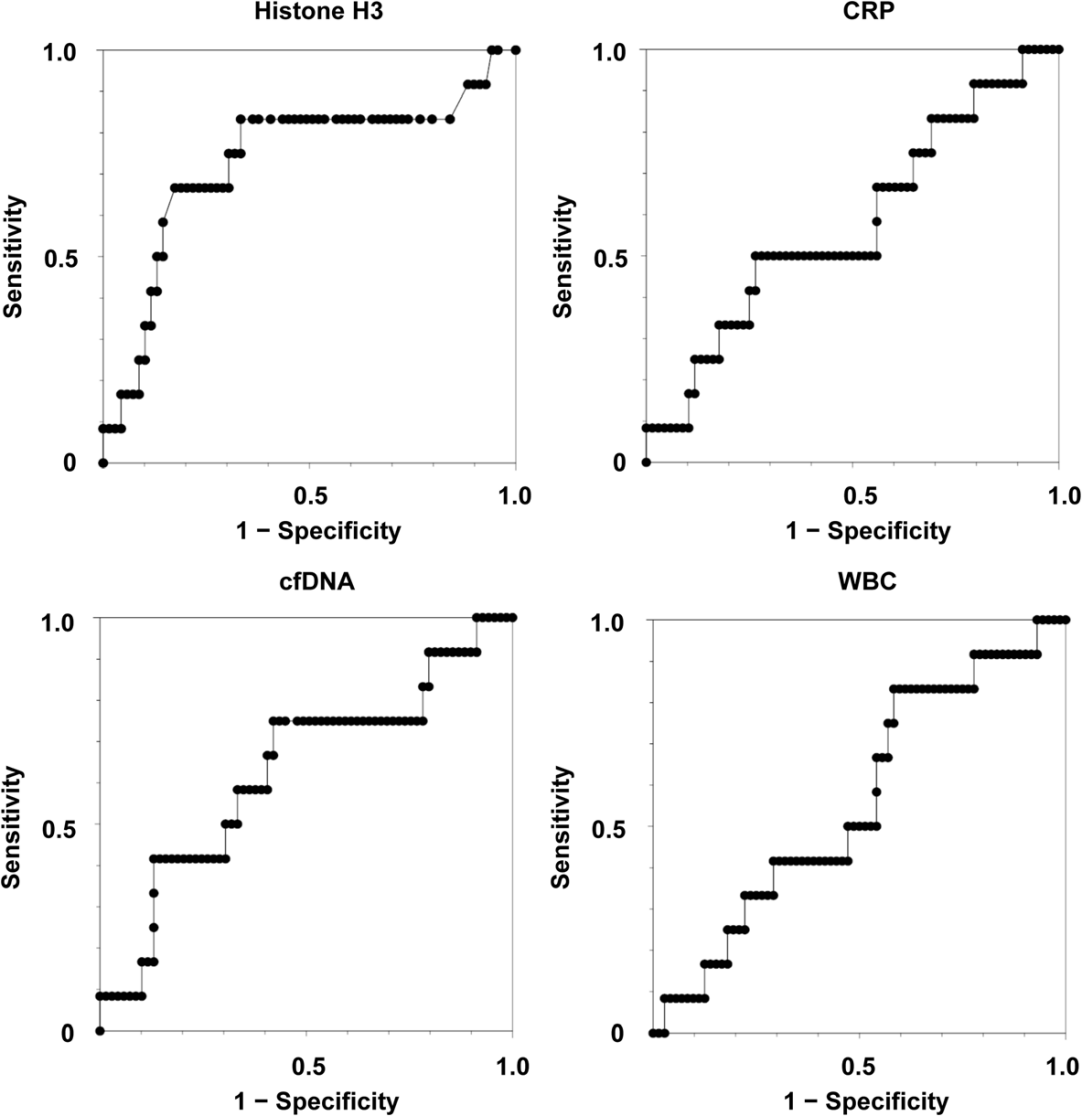
Predictive performance of circulating histone H3 levels for mortality is higher than that of conventional inflammatory markers. ROC curves for serum histone H3 levels, cell-free DNA (cfDNA) levels, CRP levels, and white blood cell (WBC) counts on day 1 are shown. The AUCs for histone H3 levels, cfDNA levels, CRP levels, and WBC counts were 0.73, 0.63, 0.58, and 0.56, respectively

The association between serum histone H3 levels and fatal outcome was evaluated (Fig. 3). Serum histone H3 levels in non-survivors (9.1 [5.1–13.0] ng/mL) were significantly higher than those in survivors (3.4 [1.5–6.1] ng/mL) at day 1. Serum histone H3 levels also tended to be higher in non-survivors on days 3, 5, and 7, but the differences did not reach statistical significance. Other conventional inflammatory markers, including serum cell-free DNA, CRP, and white blood cell counts, did not differ between survivors and non-survivors. To quantify the predictive performance of serum histone H3 levels for 28-day mortality, we analyzed the ROC curves. As shown in Fig. 4, the predictive performance of serum histone H3 levels was higher than that of the other conventional inflammatory markers. These results indicate that increased histone H3 levels are associated with fatal outcome.

## Discussion

In this study, we showed that circulating histone H3 levels were associated with coagulopathy, multiple organ failure, and death in septic patients, and could be a useful marker for recognizing the severity of sepsis. Although this study did not demonstrate a cause–effect relationship between histone H3 elevation and organ failure, previous studies indicated that histone H3 exacerbated organ failure in non-clinical settings. Circulating histones were toxic toward vascular endothelial cells [3, 8] and cardiomyocytes [5], and caused microvascular thrombosis and myocardial dysfunction [4, 9]. Histones induced platelet aggregation in vitro and thrombocytopenia in vivo [6, 7]. Furthermore, neutralizing antibodies against histones were able to rescue mice from lethal sepsis [3]. These findings indicate that high concentrations of circulating histones can be a direct inducer of organ failure, rather than a consequence of organ failure. Thus, evaluation of histone H3 levels is important for not only recognizing disease severity, but also developing strategies for histone-targeted therapy.

Circulating histone H3 levels showed a strong positive correlation with cell-free DNA levels, a modest but significant positive correlation with white blood cell counts, and no correlation with CRP levels. Circulating histone H3 levels also showed a significant positive correlation with SOFA score. The predictive performance of circulating histone H3 levels for mortality was higher than that of white blood cell counts, CRP, and cell-free DNA. However, it remains inconclusive whether circulating histone H3 levels are a better prognostic marker than cell-free DNA. Although cell-free DNA can be measured in several ways, including ultraviolet absorbance [14], DNA dyes [13, 15], and polymerase chain reaction [14, 16], the methods are not standardized. So, further studies are needed for standardization of cell-free DNA measurement and evaluation of good prognostic markers in septic conditions.

### Limitations of the study

The present study has several limitations. First, the diagnosis of sepsis was not confirmed in all patients in this retrospective study. The inclusion criteria were patients admitted to the ICU because of infectious disease. All subjects had a SOFA score of ≥2. Thus, if their SOFA scores before ICU admission (baseline SOFA scores) were assumed to be zero, all of them met the criteria for sepsis-3 [1]. However, the baseline SOFA scores were not available in this study. Second, patients who left ICU were not followed up. As a result, 73 survivors and 12 non-survivors were analyzed on day 1, while only 39 survivors and 6 non-survivors were analyzed on day 7. This may distort the composition of patients, because mild cases in the survivor group and severe cases in the non-survivor group are predisposed to leave the ICU. Third, the number of patients enrolled in this study was relatively low and did not allow us to conduct multivariate analysis. For larger scale clinical studies, the large-scale preparation of reagents, including anti-histone H3 antibodies, is required. Fourth, the specificity of circulating histone H3 levels for sepsis was yet to be investigated. It is conceivable that circulating histone H3 levels are increased in non-septic diseases, such as trauma and pancreatitis [8]. Another limitation of this study is that our ELISA did not measure other members of the histone family, including histone H4. Previous studies have shown that histone H4 is the most potent stimulator of platelets and endothelial cells, while histone H3 is the second most potent stimulator [3, 6]. The reason why we decided to measure histone H3 rather than histone H4 is the availability of good purified histone H3 as a reference standard. Further analyses of histone H4 levels and histone H3 levels may improve our understanding of the pathogenesis of sepsis.

## Conclusions

Using a newly developed ELISA for histone H3 detection, we measured the circulating histone H3 levels in patients requiring intensive care because of infectious diseases. Circulating histone H3 levels were associated with coagulopathy and multiple organ failure in these patients.

## Abbreviations

APACHE: Acute Physiology and Chronic Health Evaluation
AUC: Area under the curve
cfDNA: Cell-free DNA
CRP: C-reactive protein
ELISA: Enzyme-linked immunosorbent assay
ICU: Intensive care unit
PBS: Phosphate-buffered saline
ROC: Receiver-operating characteristic
SIRS: Systemic inflammatory response syndrome
SOFA: Sequential organ failure assessment
WBC: White blood cell
